# 

**Published:** 1896-05

**Authors:** 


### CONTENTS.

ORIGINAL ARTICLES:

Valedictory Address to the Graduates in Comparative Medicine and Veterinary Science of McGill University, Montreal. By WESLEY MILLS. M.A., M.D., D.V.S. . . . 357
The State and Veterinary Science. By R. W. HICKMAN, V.M.D. . . . 364
Milk as a Factor in the Causation of Disease. By JOHN M. PARKER, D.V.S. . 370
Osteoporosis. By GEORGE C. FAVILLE, D.V.M. . . . . . . . . 375
Heredity. By W. T. RUSSELL, V.S. . . . . . . . . . . 378
On the Use of Formalin as a Preservative Fluid. By J. T. DUNCAN, M.D. . . 383

ABSTRACTS FROM FOREIGN JOURNALS:

Tuberculosis of Monkey—Serum for Tetanus—Export of Glandered Horses—Immunizing Action of Iodide of Potassium in Regard to Aphthous Fever (Foot and Mouth Disease)—Immunity to Aphthous Fever—Inoculation of Tuberculosis of the Gallinæ in the Mammifers—The Swine-plague (Swinepest) in Austria 386-395

REPORTS OF CASES:

A Case of Canine Tuberculosis. By FRANK MILLER, V.S. . . . . . . 396
Leukæmia in a Cat. By DR. WILFRIED LELLMANN . . . . . . . 398
Myelitis in a Dog; Recovery. By ROBERT W. ELLIS, D.V.S. . . . . . 401
Urethral Calculus in a Horse. By L. A. GREINER, V.S. . . . . . . 402

CORRESPONDENCE:

Osteoporosis not Millet-disease. By DR. T. D. HINEBAUCH . . . . . 403

EDITORIAL . . . . . . . . . . . . . . . . . 406

GLEANINGS . . . . . . . . . . . . . . . . . 408

CONTROL WORK:

Massachusetts—Connecticut—Rhode Island—New York—Pennsylvania—Virginia—Michigan—Illinois—Minnesota—Iowa—Alabama—National—Army legislation—Canada . . . . . . . . . . . . . . 409-415

SOCIETY PROCEEDINGS:

McGill University, Comparative Psychology Society—U. S. V. M. A.—New York State Veterinary Medical Society—Nebraska Veterinary Medical Association—Veterinary Association of Manitoba—Massachusetts Veterinary Association—Pennsylvania State Veterinary Medical Association—Veterinary Medical Association of New York County—Wyoming Valley Veterinary Medical Association—Pennsylvania State Board of Veterinary Medical Examiners—Department of Public Safety, Philadelphia—Montreal Veterinary Medical Association . 416-427

COMMENCEMENT EXERCISES:

Ohio Veterinary College—Chicago Veterinary College—McGill University—Ontario Veterinary College—New York College of Veterinary Surgeons—United States College of Veterinary Surgeons . . . . . . . . . . 427-434

AMONG THE COLLEGES—PERSONAL—WHY WE PROGRESS . . . . 434-440

